# Effect of Caesalpinia bonduc Polyphenol Extract on Alloxan-Induced Diabetic Rats in Attenuating Hyperglycemia by Upregulating Insulin Secretion and Inhibiting JNK Signaling Pathway

**DOI:** 10.1155/2020/9020219

**Published:** 2020-03-17

**Authors:** Asra Iftikhar, Bilal Aslam, Maryam Iftikhar, Wafa Majeed, Mehwish Batool, Bushra Zahoor, Naseem Amna, Hareem Gohar, Iqra Latif

**Affiliations:** ^1^Faculty of Pharmaceutical Sciences, The University of Faisalabad, Faisalabad 38000, Pakistan; ^2^Institutes of Pharmacy, Physiology and Pharmacology, University of Agriculture, Faisalabad 38000, Pakistan; ^3^Innovation Center for Food Nutrition and Human Health, Beijing Engineering and Technology Research Center of Food Additives, Laboratory of Molecular Sensory Science, Beijing Technology & Business University, Beijing 100048, China

## Abstract

Caesalpinia bonduc has been used in herbal medicines for the treatment of a wide range of diseases from decades. The present study has explored the remedial potential and underlying mechanism of polyphenol extract of Caesalpinia bonduc in alloxanized diabetic rats. HPLC/MS analysis confirmed the presence of phenolics in considerable concentrations in Caesalpinia bonduc extract. Administration of different doses (250 and 500 mg/kg) of CPP extract to hyperglycemic rats for 8 weeks restored blood and serum glucose, insulin, glycosylated hemoglobin, leptin, amylin, and carbohydrate metabolizing enzymes level towards normal compared to alloxanized diabetic group. The effect of CPP extract on various genes such as Pdx-1, Ins-1, ngn-3, GLUT-4, and IRS-1 in insulin signaling pathway and Traf-4, Traf-6, and Mapk-8 in MAPK downstream JNK cascade was examined through qRT-PCR to access the core molecular mechanism involved in CPP-induced recovery of diabetes. Results have revealed that CPP extract reduced oxidative stress in pancreatic *β* cells by restoring free radical scavenging potential, reducing the mRNA expression of Mapk-8, Traf-4, and Traf-6, and increasing the Pdx-1, Ins-1, ngn-3, GLUT-4, and IRS-1 expression ensuing regeneration of *β* cells and subsequent insulin release from pancreas. The results obtained in this study recommend that CPP extract may be a promising therapeutic restorative agent in the treatment of diabetes mellitus.

## 1. Introduction

Diabetes mellitus is a complex metabolic disorder characterized by hyperglycemia, pancreatic beta (*β*) cells dysfunction, and abnormal lipid profile that result from metabolic deregulations, impaired insulin secretion and action, and inappropriate consumption of glucose [1]. It is one of the most prevalent chronic diseases and leads towards severe complications such as increase in production of reactive oxygen species (ROS), impairment of antioxidant enzymes [2], hyperglycemia [3], dislipidemia [4], alteration in insulin signaling pathway, and ROS-induced cellular damage [5]. All these changes will result in diabetes-associated secondary complications like nephropathy, retinopathy, neuropathy, and cardiovascular morbidity [6].

Experimental and clinical research studies have proved strong relation between oxidative stress induced by hyperglycemia and diabetes as ROS are produced in excessive amount through the oxidation of glucose [7]. Furthermore, pancreatic *β* cells are attacked by excessive ROS with the consequence of cellular damage due to weak intrinsic free radical scavenging potential [8]. Several signaling pathways are also altered by oxidative stress resulting in the release of proinflammatory cytokines, formation of advanced glycation end products (AGEs), and cell death [9]. Therefore, interference in oxidative stress has been highlighted as an important strategy for treatment of diabetes [10].

Oral antihyperglycemic agents are being used for glycemic control, but they have severe adverse effects such as abdominal pain, obesity, hepatic disorders, and renal injury [11, 12]. According to latest research, plant-derived products have demonstrated wide range of valuable therapeutic activities without causing adverse effects [11]. Plants rich in polyphenolics have gained much attention due to their wide spectrum of therapeutic benefits, as verified by both *in vitro* and *in vivo* studies [12, 13]. The polyphenols are reported to produce insulin-like effect in glucose consumption, lower ROS generation, and enhance free radical scavenging mechanism [14]. These phytoconstituents protect cellular antioxidant defense mechanism from oxidative stress, stimulate insulin signaling pathway, and regulate transcription factors, hormones, peptides, and inflammatory pathways for the management of hyperglycemic condition and diabetes-associated complications [15].

Caesalpinia bonduc (L.) Roxb. also known as “fever nut, bonduc nut and nicker nut” belongs to the family of Caesalpiniaceae and has been reported in folk medicine [16, 17]. It is a thorny perennial shrub, native of Africa, South India, Sri Lanka, Malaysia, Burma, and Ceylon, particularly along the sea coast and up to 2500 ft. in hilly areas. Caesalpinia bonduc (C. bonduc) has a wide range of therapeutic effects like antioxidant, antiviral, antianaphylactic, antipyretic, antibacterial, antidiarrheal, and antiasthamatic potential [18, 19]. These effects are due to the presence of phytoconstituents such as polyphenols, flavonoids, saponins, and terpenoids in different parts of C. bonduc such as leaves, roots, seeds, and bark. However, leaves are a rich source of polyphenol content [20]. Phenolic compounds produce antioxidant effect and reduce oxidative stress by donating hydrogen ions. Keeping in view the pharmacological activities of polyphenols, we hypothesized that polyphenols extracted from C. bonduc may improve hyperglycemic status of alloxan-induced diabetic rats through reduction/inhibition of oxidative damage and by restoration of pancreas and liver function by normalizing the activity of genes involved in insulin release and MAP kinase downstream JNK cascade.

## 2. Materials and Methods

### 2.1. The Extraction Procedure of Polyphenolics

Leaves of C. bonduc were collected from a local market and verified for taxonomy from the Department of Botany, and a voucher specimen (reference no.: 21148) was deposited in the herbarium of University of Agriculture, Faisalabad. For the preparation of extract, 500 g of C. bonduc leaves were desiccated, pulverized, and extracted with 70% ethanol. The obtained extract was kept at 25°C for 7 days. After 7 days, the mixture was filtered and the solvent was completely removed using a rotary evaporator. The residue obtained after extraction was mixed with water and extracted with petroleum ether. Treatment of residue with petroleum ether results in the formation of two separate layers. Ethyl acetate with glacial acetic acid (10 mL/L) was used to extract the aqueous layer. Organic layer was evaporated and polyphenols were extracted out from ethyl acetate layer and dried through evaporation [21]. The C. bonduc polyphenol (CPP) extract was then freeze dried (-58°C) and kept for further analysis.

### 2.2. Assessment of Total Polyphenol and Flavonoid Content of CPP Extract

The total polyphenol concentration in CPP extract was determined by the Folin-Ciocalteu (FC) colorimetric method with some modifications [21]. The absorbance was deliberate spectrophotometrically at 760 nm. Results were presented as mg gallic acid equivalents (GAE)/100 g dry weight (DW). The total flavonoid concentration was estimated by the aluminium chloride colorimetric method [22]. The absorbance was measured at 510 nm and the results were expressed as mg of (+)- catechin equivalents (CE)/100 g DW.

### 2.3. Quantification of Polyphenols in CPP Extract by HPLC-Mass Spectrometry (HPLC/MS)

The CPP extract was assayed through HPLC-MS (Agilent Technologies, USA). Different components of extract were separated using C-18 column (4.6 × 250 mm and 5 *μ*m, Agilent, USA). Mobile phase A was consisted of 2% (*v*/*v*) glacial acetic acid and B was acetonitrile. Analysis conditions were as follows: 10% B, 10 min; 25% B, 15 min; 50% B, 45 min; 75% B, 65 min; and 100% B, 75 min. The temperature of column was 28°C, flow rate was 1 mL/min and the volume of injection was 20 *μ*L. Absorbance wavelength was adjusted at 280-310 nm for the detection of phenolics using UV detector [23]. At least three independent polyphenol extractions were performed and analyzed independently by HPLC-MS. Phenolic compounds were quantified by comparing the peak area of each compound with a respective standard.

### 2.4. Experiment Animals

Male adult Wistar albino rats weighing 250 to 300 g (7-8 weeks old) were obtained from the University of Agriculture (Faisalabad, Pakistan) and kept under standard laboratory conditions (at room temperature and 12 h light-dark cycle) with *ad libitum* approach to water and pellet diet. The animals were acclimatized for a period of seven days prior to the experiment. The experiment protocol was approved by the ethical review committee (ethical review no.: 511/oric). The research study was designed and conducted in accordance with the procedures of the graduate studies research board (GSRB) and international standards for the handling of experimental animals.

### 2.5. Experimental Design

Alloxan monohydrate (120 mg/kg) mixed with normal saline (0.9% saline) was injected intraperitoneally to overnight-fasted rats except control group for the induction of diabetes. After one week, blood glucose levels of rats of all groups were observed by an Accu-Chek glucometer (Roche Diagnostic, Germany) from the tail vein. The animals with a glucose level equal to or more than 300 mg/dL were included in the study. Blood glucose levels were checked on a weekly basis for 8 weeks in order to observe the hyperglycemic status and also to examine the effects of CPP extract on alloxanized hyperglycemic rats. Rats were grouped into four groups (*n* = 15 per group) as follows:

Group 1: control receiving routine diet,

Group 2: positive control (diabetic) administered normal saline (1.5 mL/kg, ip),

Group 3: diabetic group treated with CPP extract (250 mg/kg),

Group 4: diabetic group treated with CPP extract (500 mg/kg).

After 8 weeks of experiment, the rats were fasted overnight, anaesthetized with 3% sodium pentobarbital (ip), and sacrificed. Blood samples from each rat were collected and divided into two parts. One part (about 1 mL) was kept in heparinized tubes for estimation of glycosylated hemoglobin (HbA1c), and the remaining part in nonheparinized tubes was centrifuged at 1400 g for 10 min for the separation of serum. The serum samples were separated and kept at -20°C until used for further analyses. For histopathological assessment, sections of rat's pancreas were preserved in 10% neutral buffered formalin (NBF). For gene expression analysis, rat's pancreatic and hepatic tissues were snap-frozen in liquid nitrogen, then immediately ground in a sterilized autoclaved mortar and pestle for further analysis.

### 2.6. Preparation of Tissue Homogenate

The excised tissues of some rats of all groups were homogenized with 10% (*w*/*v*) buffer (50 mM Tris-HCl, 1.15% KCl pH 7.4) and centrifuged at 9000 g for 3 min at 4°C using refrigerated centrifuge. The obtained supernatants were collected as tissue homogenate and used for the biochemical evaluation. The protein concentration of obtained homogenate was analyzed according to the procedure of [24].

### 2.7. Biochemical Examination

The fasting blood glucose level in normal, diabetic, and treated groups of rats were measured on a weekly basis during 8 weeks of experiment through the tail prick method using a glucometer. After 8 weeks of experiment, serum glucose levels of all groups were analyzed with the use of the rat glucose assay kit (81693, Crystal Chem, USA). Serum insulin levels were observed through enzyme-linked immunosorbent assay (ELISA) using the rat insulin ELISA kit (ELR-Insulin, RayBio®, Norcross, GA, USA), and the glycosylated hemoglobin level was determined by a commercially available kit (MBS2033689, My BioSure, California, USA). Serum leptin and peptide YY (PYY) levels were also assayed through ELISA kits (ab100773, Abcam, Cambridge, UK & EIAR-PYY, RayBio®, Norcross, GA, USA). The serum amylin level was determined through the rat amylin assay kit (NBP2-76735, Novus Biologicals, USA) and absorbance was deliberated at 476 nm wavelength. The liver glycogen level was assayed in accordance with the procedure of [25]. Activities of glycolytic enzymes (glucose-6-phosphate dehydrogenase and hepatic hexokinase) were measured by kits provided by Abcam (ab102529, ab136957, Abcam, Cambridge, UK), and gluconeogenic enzymes such as fructose-1,6-bisphosphatase and glucose-6-phosphatase were assayed using the commercially available ELISA kits (MBS931493 and MBS749461, My BioSure, California, USA).

### 2.8. Estimation of Intracellular ROS and Measurement of Lipid per Oxidation

Concentration of intracellular reactive oxygen species (ROS) was examined by using 2,7-dichlorofluorescein diacetate (DCF-DA) as a probe through the procedure of [26]. The development of DCF was determined with the help of a fluorescence spectrometer equipped with a FITC filter between the wavelengths of 488 to 510 nm for 10 min. Lipid peroxidation was examined in the form of malondialdehyde (MDA) using the thiobarbituric acid reactive substances (TBARS) assay kit by adopting the method of [27]. The absorbance of TBARS produced was calibrated using a spectrophotometer at 532 nm, and the results were expressed in nmol MDA/mg protein.

### 2.9. Assessment of Antioxidant Defense Activities

Superoxide dismutase (SOD) activity was accessed in hepatic and pancreatic tissue homogenates of all experimental groups of rats by the procedure of [28] and then tailored by [29]. Concentration of catalase (CAT) was determined by the procedure of [30] and expressed in *μ*mol of H_2_O_2_ decomposed/min/mg of protein. Glutathione peroxidase (GPx) enzyme level was analyzed by the procedure described by [31] and expressed in terms of nmol of glutathione oxidized/min/mg of protein. Reduced glutathione (GR) enzyme activity was examined by adopting the procedure of [32] with modifications, and the results were stated in terms of mg/dL of tissue.

### 2.10. Histopathological Examination

The pancreas of rats were dissected out after animal sacrifice, fixed in 10% neutral buffered formalin (NBF), and entrenched in paraffin. After embedding, the tissue samples were sliced with microtome into transparent and 5 *μ*m thick slices, placed on glass slide, and stained with hematoxylin and eosin for microscopic investigation [33].

### 2.11. RNA Extraction and Real-Time Quantitative PCR

RNA extraction was carried out by the TRIzol method (Thermo Fisher Scientific, Waltham, Massachusetts, USA) [34], modified according to [35] and quantified on a NanoDrop. The total isolated mRNA was reverse-transcribed to cDNA by using the RevertAid cDNA synthesis kit (Thermo Fisher Scientific) according to the manufacturer's manual. Real-time qPCR (RT-qPCR) was carried out on the iQ5 Bio-Rad machine using Maxima SYBR Green/ROX qRT-PCR Master Mix (Thermo Fisher Scientific). Expressions of the following genes were studied: Pdx-1, ngn-3, Ins-1, MapK-8, Traf-4, Traf-6, IRS-1, and GLUT-4. The reference/housekeeping gene was *β*-actin. The PCR was done for 40 cycles of two-step cDNA denaturation at 95°C for 15 seconds, annealing for 25 seconds at 52°C, and the extension time was 20 seconds at 72°C. Particular primer sequences were used for the amplification of genes (Table 1) in reaction mixture. The data was analyzed by the 2^-ΔΔCt^ method.

### 2.12. Statistical Analysis

All results were expressed as mean ± SE. Data was statistically analyzed using one-way ANOVA followed by the Duncan multiple range (DMR) test. *P* ≤ 0.05 was considered statistically significant.

## 3. Results and Discussion

### 3.1. Polyphenol Content in CPP Extract and Identification of Phenolic Compounds

Polyphenolic compounds are the most important class of phytochemicals that exhibit anti-inflammatory, antistress, and antihyperglycemic properties. The polyphenol concentration in the ethanol extract of C. bonduc was found to be 119.7 ± 3.5 mg GAE/g (Table 2). The polyphenols in the ethanolic extract of C. bonduc were separated by maceration, and the concentration of different phenolics in the extract was determined by using HPLC/MS. Gallic acid, protocatechuic acid, ferulic acid, chlorogenic acid, luteolin, p-coumaric acid, quercetin-3-methyl, caffeic acid, and epicatechin were the phenolic compounds identified in CPP extract (Table 3). Gallic acid content (597 ± 1.53 *μ*g/g) was more followed by caffeic acid (387 ± 5.16 *μ*g/g), p-coumaric acid (354 ± 2.36 *μ*g/g), chlorogenic acid (314 ± 3.53), and protocatechuic acid (302 ± 2.14) in extract. The results have identified significant (*P* ≤ 0.05) high concentrations of gallic acid and caffeic acid in CPP extract, while ferulic acid (156 ± 1.69), quercetin-3-methyl (131 ± 0.53), and luteolin (76 ± 1.03) contents were comparatively low (Table 3).

The results have shown the presence of significant (*P* ≤ 0.05) high amount of polyphenols in CPP extract. According to HPLC/MS, the concentrations of phenolic compounds were gallic acid *>* caffeic acid *>* p-coumaric acid *>* chlorogenic acid *>* protocatechuic acid *>* epicatechin*>* ferulic acid *>* quercetin-3-methyl *>* luteolin (Table 1). A lot of scientific research studies provide potential evidence about health benefits of polyphenol to humans. Particularly, gallic acid is reported to produce antioxidant effect by elevating the decreased levels of hepatic catalase enzyme and vitamin C in diabetic rats, and it also defend against damage produced by oxidative stress in hyperglycemic state [36, 37]. Caffeic acid potentially reduces weight loss, plasma glucose, and triglycerides levels and increases insulin concentration in diabetic rats [38]. Chlorogenic acid is also reported to have positive effects on glucose homeostasis, oxidative stress, inflammation, and apoptosis [39]. So the effect produced by each phenolic compound in CPP extract is responsible for the therapeutic potential of polyphenols. Thus, polyphenols protect against oxidative stress-mediated diseases. Results have also proved the potential hypoglycemic and lipid-lowering medicinal value of CPP extract.

### 3.2. Assessment of Fasting Blood Glucose, Serum Glucose, Insulin, and Glycosylated Hemoglobin (HbA1c)

**Figure 1:**
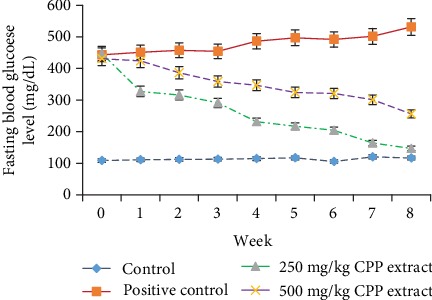
Role of CPP extract in reducing fasting blood glucose level in hyperglycemic rats.

Fasting blood glucose levels in the rats were observed weekly from week 0 to 8 throughout the experiment. According to results, there appeared a significant (*P* ≤ 0.05) rise in the glucose level after the induction of diabetes. But oral administration of CPP extract from week 1 to 8 in treated groups considerably (*P* ≤ 0.05) recovered the hyperglycemic state near to normal in both the time- and dose-dependent manners (Figure 1). Serum glucose level was also considerably (*P* ≤ 0.05) raised in all hyperglycemic rats compared to normal control group. However, treatment of diabetic groups 3 and 4 with 250 and 500 mg/kg CPP extract, respectively, significantly (*P* ≤ 0.05) reversed the upraised levels of serum glucose and insulin towards normal. The CPP treatment also significantly (*P* ≤ 0.05) ameliorated HbA1c levels towards normal compared to diabetic control rats (Table 4).

Glucose level in the body increases due to the destruction of glucose homeostasis resulting in increased HbA1c concentration and decreased level of insulin. Fasting blood glucose level is an established indicator of diabetes mellitus and its level was considerably raised in alloxanized hyperglycemic rats after the induction of diabetes. HbA1c is defined as glycation of glucose at one or more positions on the hemoglobin molecule. The International Diabetes Federation (IDF) has recommended that HbA1c is a nonmanipulatable and reliable biochemical parameter in the diagnosis of diabetes mellitus. However, fall in fasting blood glucose, serum glucose, and HbA1c levels and the improved serum insulin level predict the glucose lowering properties of the CPP extract. These results resemble previous research studies which have also identified the potent antidiabetic potential of phenolics [40].

### 3.3. Activities of Serum Leptin, Amylin and Peptide YY Levels

Table 5 shows the effect of 8 weeks CPP treatment (250 and 500 mg/kg) on the activities of serum amylin, leptin, and PYY in the diabetic group of rats. In groups 3 and 4, the serum leptin level notably declined after treatment with CPP extract compared to the positive control. Oral administrations of CPP extract also significantly (*P* ≤ 0.05) repair serum amylin activity towards normal. Moreover, the serum PYY level was appreciably reinstated after treatment of hyperglycemic rats with 500 mg/kg of CPP extract compared to 250 mg/kg.

Leptin is an important regulatory hormone of energy balance and body fat. It is an adipokine, which under normal physiological conditions functions to decrease appetite, regulate energy balance, and improve glucose consumption and insulin sensitivity [41]. Accumulating evidence indicates that leptin regulates energy expenditure by stimulating lipolysis in peripheral organs like skeletal muscle and adipose tissues and by reducing triglycerides content in liver [42, 43]. However, significant high concentration of leptin has been observed in diseased conditions such as obesity and hyperglycemia resulting in leptin resistance due to a reduced number of leptin receptors in peripheral organs. High serum leptin levels probably reflecting leptin resistance were seen in diabetic rats which predict an increased risk for diabetes. This may be because of deregulation of the adipocyte-insulin axis in pancreatic *β* cells in a state of hyperleptinemia resulting in hyperinsulinemia [44]. Anabolic action of insulin stimulates adipogenesis thereby leading to a further increase in insulin secretion and consequently insulin resistance with the development of diabetes. However, by decreasing serum leptin, CPP extract significantly improved the diabetic conditions in treated groups 3 and 4 in a dose-dependent manner. Some studies have reported a significant positive relationship between insulin resistance, diabetes, and serum leptin level [45, 46]. However, an inverse relation between serum leptin level and diabetes has also been reported [47].

PYY is a gut hormone that gets released in response to food in the ileum and colon and works to lessen hunger and to promote weight loss by slowing down gastric motility. According to results, high PYY levels in diabetic rats that may be correlated with unresponsive leptin signaling cascade as demonstrated due to leptin resistant. However, CPP extract significantly (*P* ≤ 0.05) reversed the PYY level in the diabetic group of rats. Serum amylin level was also raised to some extant in alloxan-induced diabetic rats. Accumulating evidence indicates that increased amylin level is associated with the activation of JNK cascade, production of oxidative stress, and ultimately apoptosis of *β* cells of pancreas [48, 49]. Moreover, another research study has proved that treatment of INS-1 cells with amylin significantly (*P* ≤ 0.05) increased autophagosome formation and cell death [50]. So amylin level could be considered as important target for the regeneration of *β* cells and treatment of diabetes. This argument is based on the fact that increased amylin level is connected with the apoptosis of *β* cells [51]. However, CPP extract considerably reversed the serum amylin level in treated groups. Indeed, different plant extracts have the ability to lower the amylin production [52, 53].

### 3.4. Estimation of Carbohydrate Metabolic Enzymes and Hepatic Glycogen

Table 6 demonstrates the effect of 8 weeks CPP treatment (250 and 500 mg/kg) on the levels of carbohydrate metabolizing enzymes in the liver of hyperglycemic rats. Alloxan produced considerable deterioration in the activities of glucose-6-phosphate dehydrogenase and hexokinase and a significant (*P* ≤ 0.05) decline in hepatic glycogen level, whereas a considerable rise in the level of gluconeogenic enzymes was noticed. However, treatment of alloxanized hyperglycemic rats with 250 mg/kg and 500 mg/kg CPP extract recovered the activities of gluconeogenic and glycolytic enzymes and also restored the liver glycogen content close to normal when compared with untreated control group of rats.

Glucose homeostasis depends on appropriate balance of glycolysis, glycogen metabolism, and gluconeogenesis [54]. The terminal step in hepatic glycogenolysis and gluconeogenesis is catalyzed by glucose-6-phosphatase and fructose-1, 6-bisphosphatase. These two enzymes are involved in the production of hepatic glycogen [55, 56]. Concentration of both of the enzymes was notably increased in alloxanized hyperglycemic rats. Results of our research study expressed considerable reduction in the activity of these enzymes which possibly is due to the restoration of insulin level after CPP treatment in treated groups 3 and 4 resulting in reduced glucose production. Pentose phosphate pathway also plays an important role in production of pentose sugars and reducing equivalent NADPH which are essential for maintenance of glucose homeostasis. Glucose-6-phosphate dehydrogenase is the key regulator of the initial step of pentose phosphate pathway, and its deficiency leads towards the production of ROS resulting in oxidative stress [57]. In alloxan-induced hyperglycemic rats, the activity of glucose-6-phosphate dehydrogenase was reduced compared to control. However, CPP extract appreciably (*P* ≤ 0.05) recovered the level of this enzyme and exerted protective effect against oxidative stress resulting in increased glucose utilization and improved lipogenesis due to the synthesis of fat from excess concentration of glucose.

### 3.5. Estimation of Oxidant and Antioxidant Defense Enzyme Activities

The results have summarized the effect of CPP treatment on the activity of antioxidant enzymes and lipid peroxidation level in the pancreas and liver of the control and alloxanized hyperglycemic rats. Activities of pancreatic and hepatic SOD, CAT, GR, and GPx were significantly (*P* ≤ 0.05) decreased after induction of diabetes, indicating the diminution of activities of endogenous antioxidant enzyme. However, administration of respective concentrations of CPP extract into groups 3 and 4 significantly (*P* ≤ 0.05) reinstated the antioxidant enzyme activities and considerably reduced TBARS level relative to the positive control (Tables 7 and 8). For all the biochemical parameter studied above, 8 weeks treatment of treated groups of rats with CPP (250 and 500 mg/kg) extract showed significant (*P* ≤ 0.05) effects compared to diabetic control. Alloxan is a diabetogenic agent and responsible for the production of ROS in different tissues particularly in pancreatic tissues due to its rapid uptake by pancreas [58]. That is why intracellular ROS production was measured by using DCF-DA in the hepatic tissue in our research study. According to results, alloxan significantly (*P* ≤ 0.05) raised intracellular ROS level in hyperglycemic rats. In diabetes, upraised levels of ROS may cause glucose autooxidation, inflammation, and glycosylation of protein resulting in oxidative stress [59]. The oxidative stress induces downregulation of different genes involved in insulin secretion from the pancreas leading to hyperglycemia. However, a small amount of ROS produced under normal physiological conditions plays an important role in different biological processes [60]. Hence, the concept of oxidative stress may offer a unique therapeutic option for the management of diabetes by using nutraceuticals that possess a powerful free radical scavenging capacity and ability to boost the activity of the intracellular antioxidant system [61]. The glucose lowering potential of CPP extract is credited to strong antioxidant defense produced by polyphenols resulting in the regeneration of pancreatic cellular architecture and normal insulin production and release from *β* cells.

Oxidative stress produced by the hyperglycemic state deteriorates the antioxidant defense mechanism of the body and consequently increases MDA levels and reduces the activity of antioxidant enzymes [62]. MDA, an aldehyde, is considered as a biomarker of lipid peroxidation as it is formed due to the oxidation of membrane lipids. Results had suggested that the use of CPP extract in both treated groups considerably (*P* ≤ 0.05) decreased the MDA level and raised the activities of CAT, SOD, and GPx in a dose-dependent manner. Superoxide radicals are mainly responsible for the production of ROS and oxidative stress from different sources, and their dismutation by SOD is required for proper functioning of cells [63]. Glutathione, an important antioxidant, is present in excess amount in all cells. It scavenges lipid peroxides and hydrogen peroxide of GSH-Px by providing an electron to form water and oxygen. This oxidized form is again converted into GSH through the action of GSH reductase. This reduced form of glutathione protects membrane lipids against the harmful effects of oxidants [49]. These results have identified improved the detoxification capability of CPP extract due to recovery of antioxidant enzymes towards normal resulting in protection of cellular organelles from oxidative stress. The antioxidant potential of CPP extract may be due to the restoration of the glycation of the antioxidant enzymes SOD, CAT, and GPx towards normal.

### 3.6. Histopathological Examination

**Figure 2:**
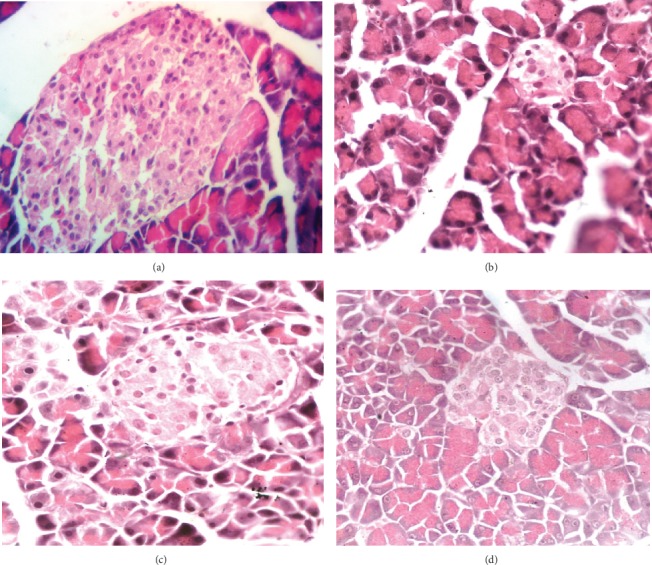
(a) Pancreatic section of the control group showed normal histological features of beta cells and islets of Langerhans. (b) Pancreatic section of the diabetic/positive control group showed degenerative and necrotic changes in the islet of the Langerhans cells, congestion, and loss of cellular content. There was atrophy and regression in the size of all of the islets of the Langerhans cells. (c, d) The responses of the treated groups (250 and 500 mg/kg CPP extract) showed active beta cells and increased dense volume of the islet cell which is a sign of regeneration.

According to the results, the pancreas of the control group showed normal histological features of the islets of the Langerhans cells with active nuclei and abundant cytoplasm (the endocrine portion) (Figure 2(a)). The pancreatic tissue of alloxan-induced diabetic rat demonstrated degeneration and inflammatory cellular infiltration, vacuolization, congestion, atrophy, massive necrotic changes, regression in the size of all *β* cells, and pycnotic nuclei compared to control group (Figure 2(b)). These histopathological changes observed in the positive control group were like those reported previously [64]. Use of 250 mg/kg of CPP extract in alloxanized hyperglycemic rats produced marked improvement in cellular injuries as evident from partial restoration of islets and *β* cells mass (Figure 2(c)). These results showed considerable improvement in the population of *β* cells and reduction in tissue necrosis. The response of the highest dose-treated group (500 mg/kg) exhibited an increased dense volume of islet cells with a reduced number of necrotic *β* cells which is a sign of regeneration (Figure 2(d)). According to the results, administration of 500 mg/kg CPP in hyperglycemic rats showed healthy architecture of islets of Langerhans with active *β* cells and absence of any necrotic change. These results are in agreement with a study in which treatment of diabetic rats with Aloe vera extracts resulted in significant increase in the number of islets of Langerhans and improvement in insulin secretion [65].

### 3.7. Effect of CPP Extract on Pancreatic and Hepatic Genes

#### 3.7.1. CPP Extract Ameliorates Traf-4, Traf-6, and MAPK-8 over Expression in Diabetic Rats

**Figure 3:**
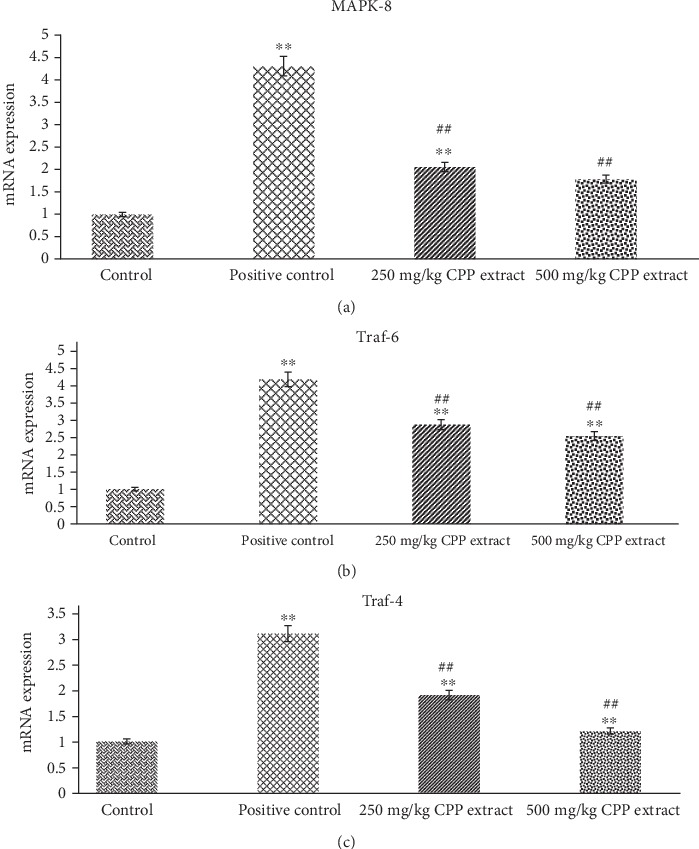
Rat's pancreatic gene expression profile: mRNA expression of (a) MAPK-8, (b) Traf-6, and (c) Traf-4 in control, positive control, 250 mg/kg, and 500 mg/kg CPP-treated groups. ^∗∗^*P* ≤ 0.01 shows significant difference between control and other groups. ^**##**^*P* ≤ 0.01 indicates significant difference between positive control and CPP-treated groups.

The MAPK downstream JNK signaling pathway has been widely confirmed to play important roles in the pathological process of diabetes. In the diabetic rats, Traf-4 and Traf-6 expressions were upregulated in response to a high level of ROS production, which significantly (*P* ≤ 0.01) differed from the normal group. Oral CPP administration (250 mg/kg) resulted in marked decline in the ROS level resulting in decreased expressions of Traf-4 and Traf-6 in the diabetic rats (*P* ≤ 0.01) which closely approximated the healthy controls. As shown in Figure 3, ROS-dependent activation of Traf-4 and Traf-6 resulted in upregulation (*P* ≤ 0.01) of MAPK-8 gene in diabetic groups relative to normal or untreated group of rats. After administration of different concentrations of CPP extract into groups 3 and 4, the phosphorylation level of MAPK-8/JNK1 was significantly decreased in different degrees. Among the data, treatment with 500 mg/kg remarkably (*P* ≤ 0.01) resisted the activation of JNK pathway resulting in significant (*P* ≤ 0.01) reduction of apoptosis of pancreatic *β* cells induced by hyperglycemia.

It has been well demonstrated that the oxidative stress-induced MAPK downstream JNK pathway plays a crucial role in the development of diabetes [66]. The JNK proteins are activated by a series of phosphorylation on threonine and tyrosine residues via MKK4 and MKK7 in response to various stress stimuli like environmental stresses and inflammatory cytokines [67]. Oxidative stress stimulates both TNF-receptor-associated factors 4 and 6 (TRAF-4) and (TRAF-6) for the activation of ASK1 and the subsequent activation of downstream JNK signaling in various tissues, including pancreatic islets [68, 69]. Upon activation, the phosphorylated JNK phosphorylates c-Jun in the nucleus as it is translocated into the nucleus upon activation. Phosphorylated c-Jun leads towards the formation of activated protein-1 (AP-1). The JNK-AP-1 pathway is reported to upregulate the expression of different proapoptotic genes involved in JNK-induced apoptosis [70].

A positive feedback effect may exist between ROS and JNK, in which increase in ROS level enhanced the expression of genes involved in JNK pathway, and activated JNK promotes the production of more ROS [71]. MapK-8, also known as Jnk-1 (c-Jun N-terminal protein kinase 1), is critically involved in the generation of oxidative stress resulting in the induction of subsequent events of stress-induced apoptosis. Furthermore, activation of Jnk-1 gene leads towards inflammation of islets of Langerhans, pancreatic *β* cells dysfunction, and release of insufficient or defective insulin from *β* cells [72]. Under diabetic conditions, the JNK pathway is stimulated in many tissues, and this activation results in insulin resistance. However, inhibition of this pathway in different research trials also resulted in the reduction of insulin resistance and on the whole diabetes [73].

In the present research study, the purpose of evaluating the expression of TRAF-4, TRAF-6, and Jnk1/MapK-8 in diabetic and control rats after CPP treatment was to study how polyphenolics improve conditions in the presence of diabetes. According to the results, expression levels of TRAF-4, TRAF-6, and MAPK-8 genes were drastically raised in hyperglycemic rats. However, CPP treatment considerably (*P* ≤ 0.01) inhibited the activation of JNK pathway induced by high glucose. Taken together, we found that CPP extract reversed the upregulation of TRAF-4 and TRAF-6 resulting in the inhibition of MAPK-8 expression. In conclusion, the present research study has confirmed the antioxidant, antihyperglycemic, and regenerative potential of CPP extract due to the significant reduction in oxidative stress, downregulation of JNK/c-Jun signaling pathway, and increase in the regeneration of pancreatic *β* cells after 8 weeks CPP treatment of alloxan-induced diabetic rats.

#### 3.7.2. Effects of CPP Extract on Ins-1, ngn-3, and Pdx-1 Expression in Diabetic Rats

**Figure 4:**
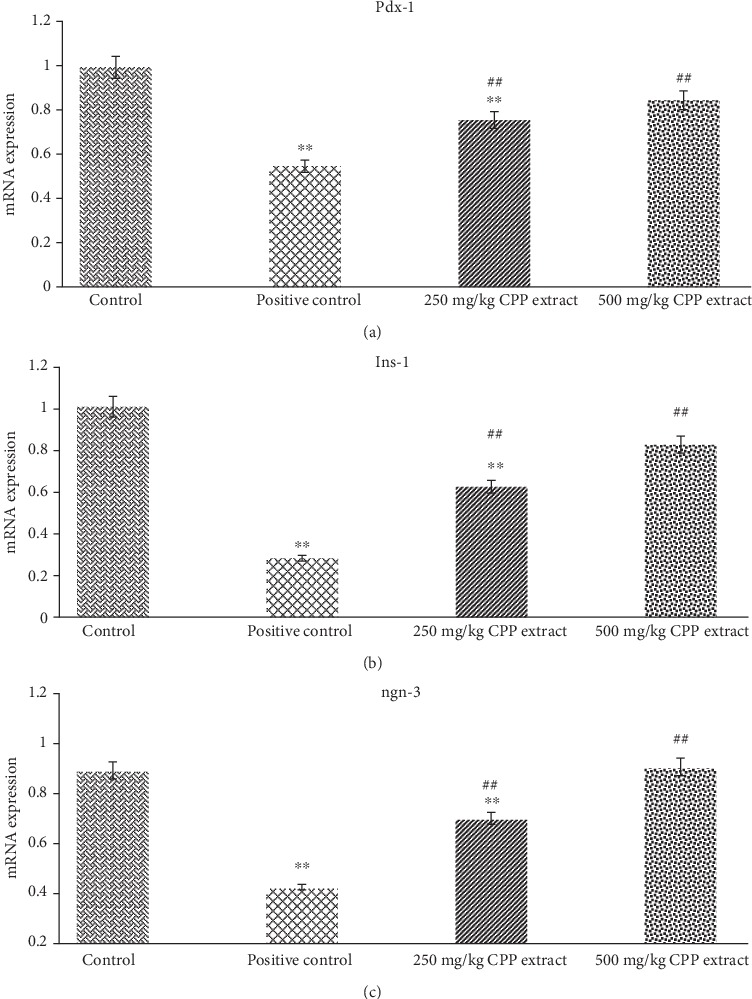
Rat's pancreatic gene expression profile: mRNA expression of (a) Pdx-1, (b) Ins-1, and (c) Ngn-3 in control, positive control, 250 mg/kg, and 500 mg/kg CPP-treated groups. ^∗∗^*P* ≤ 0.01 shows significant difference between control and other groups. ^**##**^*P* ≤ 0.01 indicates significant difference between positive control and CPP-treated groups.

In order to identify the potential mechanism adopted by CPP extract in the regeneration of *β* cells and improved insulin production, the effect of CPP on insulin signaling pathway through the expression of genes (Ins-1, ngn-3, Pdx-1, IRS-1, and Glut-4) was investigated by the RT-PCR analysis. Expression levels of Ins-1, ngn-3, and Pdx-1 in pancreatic tissues were appreciably (*P* ≤ 0.01) downregulated in the diabetic rats compared with the normal group. The results have explored that CPP extract significantly (*P* ≤ 0.01) increased Ins-1, Pdx-1, and ngn-3 protein expression resulting in the regeneration of *β* cells of pancreas (Figure 4).

#### 3.7.3. Effects of CPP Extract Treatment on IRS-1 and Glut-4 Genes Level in Target Rats

**Figure 5:**
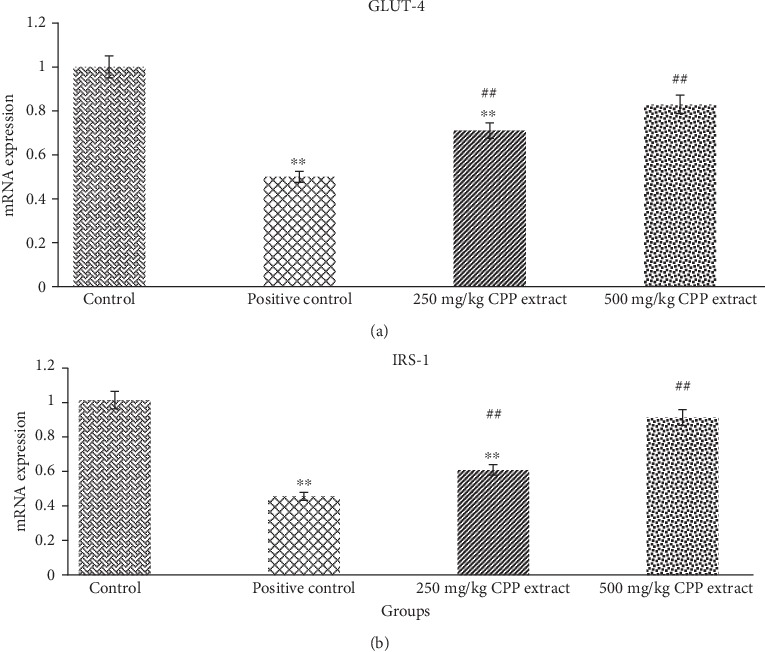
Rat's hepatic gene expression profile: mRNA expression of (a) GLUT-4 and (b) IRS-1 in control, positive control, 250 mg/kg, and 500 mg/kg CPP-treated groups. ^∗∗^*P* ≤ 0.01 shows significant difference between control and other groups. ^**##**^*P* ≤ 0.01 indicates significant difference between positive control and CPP-treated groups.

To investigate the mechanisms of CPP extract on insulin signaling pathway, expression levels of different genes including IRS-1 and Glut-4 were investigated. The transcription levels of IRS-1 and Glut-4 genes were examined in the liver samples of control, diabetic, and CPP-treated groups. According to the results, IRS-1 and Glut-4 gene expression was significantly (*P* ≤ 0.01) reduced in the positive control group of rats. However, oral administration of CPP extract (250 mg/kg and 500 mg/kg) in treated groups 3 and 4 of rats considerably (*P* ≤ 0.01) increased the levels of IRS-1 compared with the model group. In addition, CPP treatment also significantly (*P* ≤ 0.01) promoted GLUT-4 protein expression resulting in the improvement of insulin resistance compared to positive control group (Figure 5).

As shown in Figures 4 and 5, all genes were downregulated in diabetic rats. After CPP treatment, gene expression levels were significantly (*P* ≤ 0.01) restored towards normal, which indicates improvement in *β* cells efficiency and alleviation of insulin resistance in all insulin-affected tissues. Activation of JNK pathway leads to phosphorylation of serine residue at particular sites in insulin receptor substrate-1 (IRS-1), which decreases the potential of IRS-1 to transfer the signals [74]. The delivery of insulin signals begins with phosphorylation of tyrosine residue at insulin receptor and its substrate [75]. The CPP extract could increase IRS-1 protein phosphorylation, activate PI3K/AKT pathway, stimulate GLUT-4 translocation to the cell membrane, and enhance glucose transport and lipid metabolism [76, 77].

The pancreatic and duodenal homeobox 1 (Pdx-1) is an important gene involved in the development and functioning of *β* cells [72]. Similar to MafA, it is regarded as a key controller and regulator of glucose-stimulated insulin gene (Ins-1) transcription. The neurogenin-3 (ngn-3) is important in the central nervous system development and cell differentiation of pancreatic *β* cells [78]. Both of these transcription factors, ngn-3 and Pdx-1, are markers of endocrine cell precursors. Beta cell damage caused by either oxidative stress or glucotoxicity downregulates the expression of Pdx-1, and downregulation of Pdx-1 significantly (*P* ≤ 0.01) reduces insulin production favoring induction of diabetes. Moreover, downregulation of ngn-3 at embryonic stages results in undifferentiated endocrine part of the pancreas [79]. Indeed, in our research study, activation of JNK pathway in diabetic rats drastically inhibited expression of ngn-3 and nuclear localization of Pdx-1 and its DNA binding capability resulting in impaired insulin gene (Ins-1) expression. Administration of 250 mg/kg and 500 of CPP extract in treated groups of rats appreciably (*P* ≤ 0.01) improved the Pdx-1 and ngn-3 gene expressions resulting in the regeneration of *β* cells and increased insulin release from *β* cells.

## 4. Conclusion

The present study suggests that CPP confers antihyperglycemic effect on alloxanized diabetic rats by reducing oxidative stress and downregulating the expression of different genes involved in JNK pathway. The CPP extract has also ameliorated diabetes-induced pancreatic *β* cells loss by repairing the antioxidant defense mechanism and restoring insulin, amylin, leptin, and carbohydrate-metabolizing enzyme levels. Moreover, CPP treatment has protective effects on the structure and function of *β* cells which may be accompanied by the upregulation of genes involved in insulin signaling pathway.

## Figures and Tables

**Table 1:** Primer sequences of genes used for RT-qPCR.

Gene number/name	Primer sequence (5′ to 3′)	Direction
Traf-6	GCGCCTAGTAAGACAGGACC	Forward
	CACATGCATGCTCTGCGTTT	Reverse

Mapk-8	CTCAGCATCCGGTCTCTTCG	Forward
	CTGCTGTCTGTATCCGAGGC	Reverse

Traf-4	CGACTACAAGTTCCTGGAGAAGC	Forward
	AGGTGTCGCAGAAGCGGTG	Reverse

Pdx-1	TCCCGAATGGAACCGAGACT	Forward
	TTCATCCACGGGAAAGGGAG	Reverse

Ins-1	AGGCTCTGTACCTGGTGTGTG	Forward
	CGGGTCCTCCACTTCACGAC	Reverse

Ngn-3	TGCAGCCACATCAAACTCTC	Forward
	GGTCACCCTGGAAAAAGTGA	Reverse

IRS-1	GATACCGATGGCTTCTCAGACG	Forward
	TCGTTCTCATAATACTCCAGGCG	Reverse

GLUT-4	ACTCTTGCCACACAGGCTCT	Forward
	AATGGAGACTGATGCGCTCT	Reverse

Beta action	CGAGTACAACCTTCTTGCAGC	Forward
	TATCGTCATCCATGGCGAACTG	Reverse

**Table 2:** Total polyphenol and total flavonoid concentration present in CPP extract.

Total polyphenols (mg GAE/g sample)	Total flavonoids (mg CE/g sample)
119.7 ± 3.5	27 ± 2.1

GAE = gallic acid, CE = catechin.

**Table 3:** Concentration of phytoconstituents present in CPP extract.

Phenolic constituent	*μ*g/g dry extract
Gallic acid	597 ± 1.53
Protocatechuic acid	302 ± 2.14
Chlorogenic acid	314 ± 3.53
Ferulic acid	156 ± 1.69
Caffeic acid	387 ± 5.16
Luteolin	76 ± 1.03
Quercetin-3-methyl	131 ± 0.53
p-Coumaric acid	354 ± 2.36
Epicatechin	257 ± 1.47

Data presented as mean ± SE (*n* = 3).

**Table 4:** Effect of CPP treatment on serum glucose, insulin, and glycosylated hemoglobin level in diabetic rats after 8 weeks.

Parameters	Groups			
	Normal	Positive control	250 mg/kg CPP extract	500 mg/kg CPP extract
Serum glucose (mg/dL)	113.70 ± 4.28D	497 ± 4.81A	218.46 ± 2.71B	134.56 ± 2.09 BC
Serum insulin (mg/dL)	19.5 ± 0.73A	7.37 ± 0.93D	13.54 ± 0.58 BC	16.64 ± 0.75 BC
HbA1c (%)	5.68 ± 0.05E	11.25 ± 0.11A	8.74 ± 0.09C	67.01 ± 0.06D

Data presented as mean ± SE. Values not having the same letters show significant difference within a column (*P* ≤ 0.05).

**Table 5:** Effect of CPP treatment on serum leptin, amylin, and peptide YY levels in diabetic rats after 8 weeks.

Parameters	Groups			
	Normal	Positive control	250 mg/kg CPP extract	500 mg/kg CPP extract
Serum amylin (ng/mL)	58.3 ± 5.82C	76.5 ± 6.21A	67.7 ± 5.36 B	61.2 ± 5.09BC
Serum leptin (ng/mL)	0.91 ± 0.08C	2.9 ± 0.61A	1.87 ± 0.53B	1.22 ± 0.47BC
PYY (pg/mL)	37 ± 2.31CD	170 ± 4.11A	81.5 ± 3.87B	52.7 ± 3.21BC

Data presented as mean ± SE. Values not having the same letters show significant difference within a column (*P* ≤ 0.05).

**Table 6:** Effect of CPP treatment on carbohydrate metabolizing enzymes and glycogen level in the liver of diabetic rats.

Parameters	Groups			
	Normal	Positive control	250 mg/kg CPP extract	500 mg/kg CPP extract
Hepatic hexokinase (*μ*mol of glucose-6-PO_4_ formed/min/mg protein)	268 ± 11.45A	108.63 ± 9.6D	169.56 ± 7.8C	241.51 ± 8.6B
Gluocse-6-phosphatase (*μ*mol of Pi liberated/min/mg protein)	0.16 ± 0.02BC	0.27 ± 0.06A	0.20 ± 0.04B	0.17 ± 0.43BC
Fructose 1-6-biphosphatase (*μ*mol of Pi liberated/min/mg protein)	0.65 ± 0.07BC	0.81 ± 0.04A	0.76 ± 0.035AB	0.69 ± 0.09B
Glucose-6-phosphate dehydrogenase (U/min/mg protein)	3.67 ± 0.97A	1.54 ± 0.45BC	2.01 ± 0.57B	3.37 ± 0.76A
Liver glycogen (mg/g tissue)	47.3 ± 2.6A	23.5 ± 1.97BC	36.41 ± 2.1B	42.35 ± 1.85AB

Data presented as mean ± SE. Values not having the same letters show significant difference within a column (*P* ≤ 0.05).

**Table 7:** Effect of CPP treatment on hepatic antioxidant enzymes levels in diabetic rats after 8 weeks.

Parameter	Normal	Positive control	250 mg/kg CPP extract	500 mg/kg CPP extract
Catalase (*μ*mol H_2_O_2_/min/mg protein)	53 ± 2.03A	29.5 ± 1.36CD	36 ± 2.05BC	47.5 ± 1.74B
GPx (nmol/min/mg protein)	24 ± 1.79A	14 ± 0.95C	19 ± 1.04BC	21.5 ± 1.34AB
SOD (U/mg proteins)	6.3 ± 1.09A	3.8 ± 0.84C	4.7 ± 1.36B	5.9 ± 1.03AB
GSH (mg/dL tissue)	35 ± 1.76A	24 ± 1.2C	29 ± 1.64BC	32.5 ± 1.58AB
ROS (%)	98 ± 3.6CD	184 ± 4.3A	136 ± 2.8B	107 ± 2.3C
TBARS (nmol MDA/mg protein)	2.77 ± 1.1BC	5.6 ± 1.06A	4.32.5 ± 0.93AB	3.15 ± 0.84B

Data presented as mean ± SE. Values not having the same letters show significant difference within a column (*P* ≤ 0.05).

**Table 8:** Effect of CPP treatment on pancreatic antioxidant enzymes level in diabetic rats after 8 weeks.

Parameter	Normal	Positive control	250 mg/kg CPP extract	500 mg/kg CPP extract
Catalase (*μ*mol/min/mg protein)	37 ± 1.9A	18.45 ± 1.02C	23.5 ± 1.27B	32.48 ± 2.2AB
GPx (nmol/min/mg protein)	17.5 ± 1.5A	9.5 ± 0.98C	12.38 ± 1.02B	15.87 ± 1.64AB
SOD (U/mg proteins)	4.1 ± 0.87A	2.4 ± 0.64C	3.05 ± 0.78B	3.8 ± 0.84AB
GSH (mg/dL tissue)	28.1 ± 0.9A	16.18 ± 1.04D	19.5 ± 0.86C	24.5 ± 1.3B
TBARS (nmol MDA/mg protein)	2.21 ± 1.04C	4.13 ± 1.01A	3.52 ± 0.87B	2.86 ± 0.79C

Data presented as mean ± SE. Values not having the same letters show significant difference within a column (*P* ≤ 0.05).

## Data Availability

The data used to support the results of present research work are accessible and can be obtained from the corresponding author on demand.
